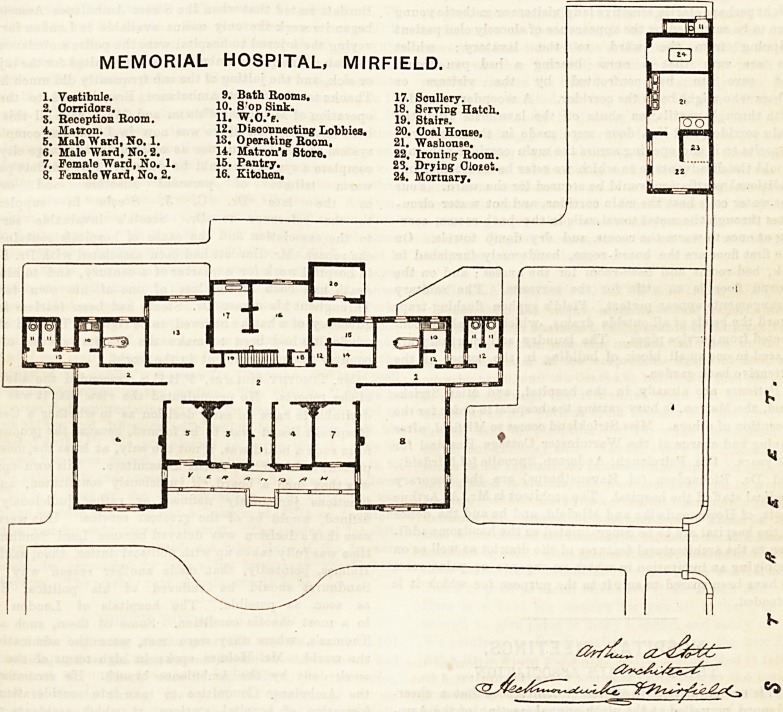# The Memorial Hospital, Mirfield

**Published:** 1893-08-12

**Authors:** 


### The Institutional Workshop.

### HOSPITAL CONSTRUCTION.

### THE MEMORIAL HOSPITAL, MIRFIELD.

This hospital, built by Mr. Charles Wheatley as a memorial of a deceased sister, provides accommodation for sixteen patients, and has cost, inclusive of site and furniture, £7,300. This is at the rate of £456 per bed; but it should be stated that a large, handsome room, now used as a board-room, can, if necessary, be converted into a ward, and that the necessity of making a road to the gates of the hospital has added to the cost of construction. Mr. Wheatley, in addition to proiding and equipping the hospital, has promised an endowment of £100 a year towards the working expenses. The hospital, a handsome building of freestone facing due south, stands on an eminence overlooking the busy valley in which Mirfield lies. Yet so well has the site been chosen that green fields and trees, ending in the woods which climb the hills on the opposite side of the valley, almost completely blot out the kilns, mills, and long chimneys which here abound, and the surrounding atmosphere is clear and bright. The hospital is built upon a plateau raised within its spacious grounds, and consists of a central tapering, three-storey building, from which a wing, projecting slightly forwards, springs at each end. A verandah, covering an open tiled promenade provided with seats, runs the length of the central portion in the recess formed by the projection of the end wings. On the ground floor a corridor runs the length of the building, and round this corridor the hospital is built. Entering at the front we note the carved oak doorway, the pretty coloured dado of ornamental tiles in the lobby, and the oaken hat-stand, before we reach the marble mosaic floor and white tiled dado of the main corridor. To the left of the entrance is the doctor's room, to the right the Matron's; then on each side an isolation ward of one bed, with a cubic space of 2,346 feet, and then the general ward at each end. On the other side of the corridor are kitchen; scullery fitted with one of Marsh's new stoves; operation-room and surgery, with marble mosaic floor, lantern light, as well as window facing due north; store-rooms, w.c.'s, bath-rooms, and lavatories. The general wards, one male and one female, are alike in size and arrangement. They are intended for seven patients each, with a cubic space of 1,426 feet per bed. The floors are pitch pine, laid over concrete, with an air space between. The walls are Parian cement, covered with Aquol paint. Shorland's patent stoves are fitted throughout the hospital, those in the general wards being fixed in the centre of the room, with descending flue. The windows are the ordinary sash windows, worked by Meakin's sash openers. The two general wards are well-lighted rooms in which there will be plenty of sunlight, and when the hospital is in working

### MEMORIAL HOSPITAL, MIRFIELD.

1. Vestibule.

2. Corridors.

3. Reception Room.

4. Matron.

5. Male Ward, No. 1.

6. Male Ward, No. 2.

7. Female Ward, No. 1.

8. Female Ward, No. 2.

9. Bath Rooms.

10. Slop Sink.

11. W.C.'s.

12. Disconnecting Lobbies.

13. Operating Room.

14. Matron's Store.

15. Pantry.

16. Kitchen.

17. Scullery.

18. Serving Hatch.

19. Stairs.

20. Coal House.

21. Washouse.

22. Ironing Room.

23. Drying Closet.

24. Mortuary.

order, when the floors have been polished, and pictures, plants, with perhaps a canary, have been added, will be bright cheerful rooms. The chairs, tables, cupboards and bedside lockers, like the furniture throughout the hospital, are of oak; and an electric wire with detachable button reaching to the patient's hands at the head of each bed enables each patient to summon the nurse or Matron at any moment. A central chandelier with ventilation apertures in the ceiling provides light. One feature calls for comment. The water closets and lavatory of each ward are placed apart on the other side of the main corridor. The ward projects into the main corridor, narrowing it at the end, and is entered by a door which looks not across but down the corridor. Consequently to go from the ward to the lavatories, which are excellently arranged, it is necessary to go into the main corridor and round the projecting corner of the ward, and it might perhaps startle sensitive lady visitors or æsthetic young men to be surprised by the appearance of a loosely clad patient tripping from the ward to the lavatory; whilst we are sure that a nurse bearing a bed pan would not care to be confronted by the visitors or others who might be in the corridor. A secondary corridor with through ventilation shuts off the lavatories from the main corridor, and if a door were made in the ward wall opposite to it and opening across the main corridor not only would the disadvantage to which we refer be overcome, but additional ventilation would be secured for the ward. Four hot-water coils heat the main corridor, and hot water circulates through the metal towel-rails in the bath-rooms, serving at once to warm the rooms, and dry damp towels. On the first floor are the board-room, handsomely furnished in oak, bed-rooms and bath-room for the nurses; and on the second floor is an attic for the servants. The sanitary arrangements appear perfect. Field's syphon flushing-traps guard the heads of all outside drains, which are all disconnected from service pipes. The laundry and mortuary are placed in one small block of building in the corner of the extensive back garden.

Patients are already in the hospital, and Miss Strickland, the Matron, is busy getting the hospital in order for the reception of others. Miss Strickland comes to Mirfield, after having had charge of the Warminster Cottage Hospital for six years. Drs. Fairclough, Anderson, Sproulle (of Mirfield), and Dr. Richardson (of Ravensthorpe) are the honorary medical staff of the hospital. The architect is Mr. A. Arthur Stott, of Heckmondwike and Mirfield, and he and the donor of the hospital are to be congratulated on the handsome addition to the architectural features of the district as well as on supplying an institution in which no expense or pains seem to have been spared to suit it to the purpose for which it is intended.

## Figures and Tables

**Figure f1:**